# Comparative Study of Neural Network Frameworks for the Next Generation of Adaptive Optics Systems

**DOI:** 10.3390/s17061263

**Published:** 2017-06-02

**Authors:** Carlos González-Gutiérrez, Jesús Daniel Santos, Mario Martínez-Zarzuela, Alistair G. Basden, James Osborn, Francisco Javier Díaz-Pernas, Francisco Javier De Cos Juez

**Affiliations:** 1 Mining Exploitation and Prospecting Department, University of Oviedo, 33004 Oviedo, Spain; gonzalezgcarlos@uniovi.es (C.G.-G.); fjcos@uniovi.es (F.J.d.C.J.); 2 Department of Physics, University of Oviedo, 33004 Oviedo, Spain; 3 Department of Signal Theory and Communications and Telematics Engineering, University of Valladolid, 47011 Valladolid, Spain; mario.martinez@tel.uva.es (M.M.-Z.); pacper@tel.uva.es (F.J.D.-P.); 4 Department of Physics, Centre for Advanced Instrumentation, University of Durham, South Road, Durham DH1 3LE, UK; a.g.basden@durham.ac.uk (A.G.B.); james.osborn@durham.ac.uk (J.O.)

**Keywords:** adaptive optics, neural networks, tomographic reconstructor, parallel processing

## Abstract

Many of the next generation of adaptive optics systems on large and extremely large telescopes require tomographic techniques in order to correct for atmospheric turbulence over a large field of view. Multi-object adaptive optics is one such technique. In this paper, different implementations of a tomographic reconstructor based on a machine learning architecture named “CARMEN” are presented. Basic concepts of adaptive optics are introduced first, with a short explanation of three different control systems used on real telescopes and the sensors utilised. The operation of the reconstructor, along with the three neural network frameworks used, and the developed CUDA code are detailed. Changes to the size of the reconstructor influence the training and execution time of the neural network. The native CUDA code turns out to be the best choice for all the systems, although some of the other frameworks offer good performance under certain circumstances.

## 1. Introduction

One of the biggest issues for the observation of astronomical targets using ground-based telescopes is the optical aberration produced by the Earth’s atmosphere in the received images. This problem can be overcome with the use of adaptive optics (AO) [1]. An AO system works by measuring the wave-front of the incoming light with a wave-front sensor and correcting it by means of a computer controlled deformable mirror. This correction has to be applied in a very short time to freeze the optical atmospheric aberrations. The light coming from guide stars is used as a reference source from which the desired deformable mirror shape is computed using a real-time control system [2]. These guide stars can be natural or artificial stars created by Rayleigh laser scattering or sodium fluorescence in the upper atmosphere. For wide-field AO systems the error introduced by the atmospheric turbulence can be measured in different directions and computer tomography techniques can be used to calculate the aberration in the direction of the object of interest. This allows for the real-time compensation of the astronomical image with the deformable mirrors [3].

The creation and development of algorithms that allow us to reconstruct the deformations introduced by the Earth’s atmosphere with the highest possible accuracy is one of the main challenges in wide-field AO. The most commonly used reconstructor is based on a matrix vector multiplication operation, with the control matrix being defined by either least squares [4,5] or minimum variance techniques [6]. In recent years, success has also been had using the Learn and Apply (L&A) method [7] and the Complex Atmospheric Reconstructor based on Machine lEarNing (CARMEN), which has shown interesting and promising results [8].

Modern wide-field AO systems for large telescopes require tomographic techniques to reconstruct the phase aberrations induced by the turbulent atmosphere at different heights above the telescope [9]. Multi-object adaptive optics (MOAO) is one of these techniques [10,11]. CARMEN is a MOAO reconstructor created at the University of Oviedo. It was initially developed using regression techniques such as multivariate adaptive regression splines (MARS) [12,13], with promising results [3,14]. However, the use of machine learning techniques such as Supporting Vector Machines (SVM) [15,16,17,18] or Artificial Neural Networks (ANN) have proven very successful in different fields [19,20], which led us to create a solution based on an ANN [21]. The ANN is trained with a large range of possible turbulent layer positions (heights) by artificially introducing turbulence at a single atmospheric conjugate height during training, and then repeating the turbulence for many different heights. Because the ANN is trained in this way, it does not require knowledge of the actual optical turbulence height distribution profile when it is in operation, rather, it learns to identify the main turbulence heights from the wave-front sensor information. The non-linear response of the ANN makes the reconstructor more robust with noisy wave-front measurements than other linear techniques. CARMEN has shown promising results in on-sky testing [22,23].

The development of large telescopes, in particular the future European Extremely Large Telescope (E-ELT), creates an extraordinary challenge in the AO field not least in terms of the computational capability needed to operate the real-time control system [24]. Due to the large number of subapertures and guide stars involved, tomography on ELT scales becomes increasingly computationally expensive. The strength of the ANN for tomographic reconstruction comes from its non-linear properties. Unfortunately, ANNs require very high computational times, but they can be highly parallelised. The use of Graphics Processor Units (GPUs) provides a solution to this problem. Due to the parallelization of different calculations, the processing time can be significantly reduced. In recent years, there have been some initiatives to adapt existing tomographic reconstructors to GPUs [25], such as the Learn and Apply case [26], and a first version of CARMEN [27,28].

During the past few years, there have been increasing numbers of software libraries and frameworks that help developers to easily train and execute ANNs [29]. Those focused on GPU have been very successful. Although there are some informal studies comparing the performance of some of these frameworks [30,31,32], to the best of our knowledge, there is no previous work that also compares them against a neural network programmed in CUDA native code. No information about frameworks that include similar network architectures to CARMEN has been found. It is, therefore, interesting to make a comparison of the implementation of the reconstructor using different frameworks, and also compare their performance against our ANN developed using CUDA.

The main purpose of this paper is to detail the implementation of CARMEN in different neural network frameworks based on GPU, and compare their training and execution times. In Section 2 we show how AO systems work, and in Section 3 the architecture of CARMEN is explained as well as the different neural networks frameworks used. The experiment is defined and different variables to compare different frameworks are proposed. Finally, results are analysed and conclusions are put forward.

## 2. Adaptive Optics Systems 

The Shack-Hartmann Wave-front Sensor (SHWFS) [33] is commonly used in astronomy to characterize an incoming wave-front. It is composed of a grid of lenses (called lenslets) which have the same focal length. Each one of them are focused on separated photon sensors, typically a charge-coupled device (CCD). By using the SHWFS, it is possible to divide the wave-front in discrete sub-pupils, where the deviation from the focal spot can be measured due to the local wave-front tilt. The information is used to create a vector of tilts (or wave-front gradients), which characterizes the wave-front aberration.

**Figure 1:**
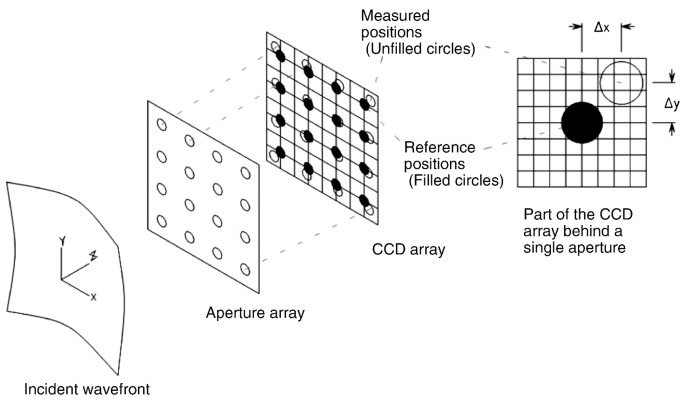
Measurement of Wave Front Tilts using a Shack-Hartmann Wave-front Sensor (SHWFS), reproduced from [38].

As shown in Figure 1, from each subaperture of the lenslet array, information about the light dispersion caused by the atmosphere can be obtained. Using this information, the shape of the wave-front using Zernike Polynomials can be reconstructed, which allows for the comparison of the difference between incident wave-front and deformable mirror surface shape. The residual wave-front error (i.e., the resulting wave-front) defines the performance of the AO system, and the root-mean square wave-front error (WFE) can be computed [34]

MOAO systems, such as CANARY [35,36], use Shack Hartman sensors to retrieve the aberration information from different guide stars, which can be positioned at any place inside the field of view of the system. By combining the information of the different reference stars, it is possible to estimate the wave-front aberrations using tomographic reconstruction techniques. There are multiple ways to reconstruct the wave-front using the information of different guide stars as sources. These may involve inverting a matrix, solving Poisson’s equation subject to certain boundary conditions, or using the neural networks based algorithm described later in this article [37].

**Figure 2:**
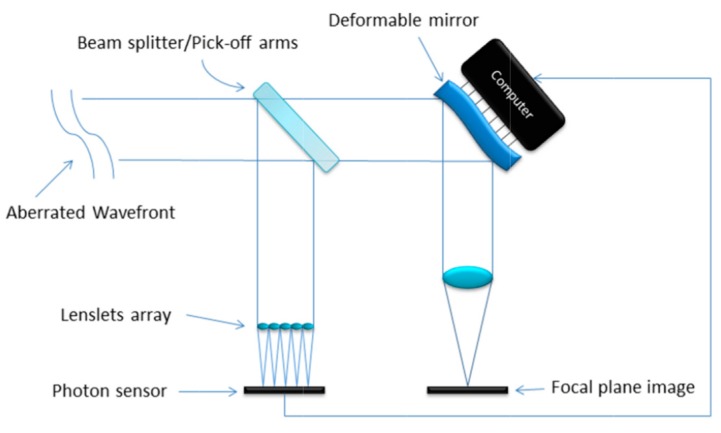
Adaptive optics loop, reproduced from [8].

After the computation of the wave-front perturbations caused by the turbulent atmosphere, a deformable mirror is shaped to compensate the incoming wave-front. Figure 2 is a simplification of how an open loop adaptive optics system works. The aberrated wave-front goes through the telescope and is split into two paths by a beam splitter. One path is deviated towards the SHWFS, and so is used to measure the wave-front perturbations. The second path goes to the deformable mirror, where the aberration is compensated, providing a flatter wave-front and a better image for observation. In an open-loop system such as this, the wave-front sensor is not sensitive to changes applied by the deformable mirror. Therefore, a high quality deformable mirror with a well characterized response and no hysteresis is required. 

CANARY [39] is an adaptive optics (AO) on-sky demonstrator, principally intended for developing and testing AO concepts for the future 39 m European Extremely Large Telescope. It is operated on a Nasmyth platform of the 4.2 m William Herschel Telescope, one of the Isaac Newton Group of Telescopes (ING) of the *Observatorio del Roque de los Muchachos* (ORM), La Palma, Canary Islands, Spain.

CANARY has operated in several configurations to demonstrate different AO techniques on-sky [35]. Different sizes and improved SHWFS have been used through the years, which provides a wide range of configurations. Here, we are going to focus on two of them, which were operated during several nights in 2013 and 2015 [35]:

- **CANARY Phase B1**: is designed to perform observations with one Rayleigh Laser Guide Star (LGS), and up to four Natural Guide Stars (NGSs). It has a Shack Hartman Wavefront Sensor with 7 × 7 subapertures, although only 36 of them are activated due to the circular telescope pupil and secondary obscuration.
- **CANARY Phase C2**: is designed for the study of Laser Tomography AO (LTAO) and Multi-Object AO (MOAO). There are four Rayleigh Laser Guide Stars, and the corresponding wave-front sensors have 14 × 14 subapertures (144 active).

We will also present results developed on the DRAGON AO laboratory at Durham University. This system has eight wave-front sensors, each with 30 × 30 sub-apertures, and, therefore, has significantly higher complexity than CANARY and is expected to be a reference in AO testing during the next few years [40].

- **DRAGON**: DRAGON aims to replicate CANARY concepts, to provide a single channel MOAO system with a woofer-tweeter DM configuration, four NGSs and four LGSs each with 30 × 30 subapertures. In this case, DRAGON is still a prototype, so we are going to use the most challenging case scenario where all the subapertures are functional, which gives as a total of 900 subapertures per star.

It could be interesting to include as a comparison a neural network with similar size to that which is expected of the forthcoming E-ELT, which will have about 100 k inputs and 5 k outputs (for each MOAO channel) [41]. However, a network that large, requires an enormous amount of VRAM, due to the size of the weighting matrices. The studied frameworks are not ready to handle that amount of data in a single GPU, so we focused only on AO systems which can fit in one GPU.

## 3. CARMEN Architecture

CARMEN is a tomographic reconstructor based on artificial neural networks whose architecture is a multi-layer perceptron with a single hidden layer. It is composed of two fully-connected layers, where each neuron is connected to all the neurons in the previous layer. The output of each neuron follows Equation (1), where *w* is the weight of each connection, *x* is the value of the neurons in the previous layer, *b* is a constant value called *bias*, and f is an activation function.

(1) $$Y=f\left(\overset{n}{\underset{i=0}{\sum }}\left(w_{i}\cdot x_{i}\right)+b\right)$$

It is not the purpose of this paper to analyse the net size, since it has been previously tested [42,43], so the number of neurons in the hidden layer will be equal to the number of neurons in the input layer. The number of input, hidden, and output neurons is directly related to the optical instrumentation used since it changes with the number of subapertures in the wave-front sensor. Also, the number of input and hidden neurons depends on the number of stars (both natural and artificial) that we observed. This means that the size of the network is highly variable and should be adapted to each asterism. However, and to make it easier to compare, used the three configurations explained in the previous section and assumed that we observed both natural and laser guide stars.

**Figure 3:**
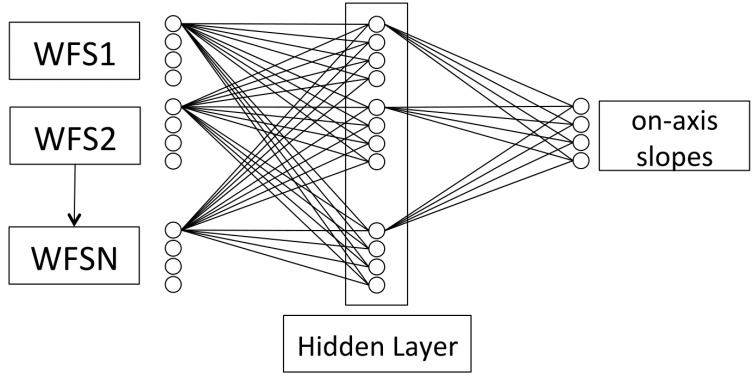
CARMEN architecture, reproduced from [22].

These rules mean that the number of input neurons matches the number of functional sub-apertures multiplied by 2 (due to the two-dimensional input in the lenslet array) and also multiplied by the number of reference stars. The output of the ANN is the expected wave-front slope measurements as seen by the on-axis wave-front sensor and has a size equal to twice the number of sub-apertures. The final topology of the network is summarized in Figure 3.

A network with 1 LGS and 2 NGSs was used in the case of CANARY Phase B1, that is, 216 input neurons. For CANARY Phase C2, with four Laser Guide Stars, there were 1152 input neurons. In both cases, the training data was obtained from the CANARY simulator. For these tests, it is preferable to use computer or bench simulated data instead of the information provided from a real telescope, since it is easier to control all the parameters required for training the network.

Regarding DRAGON, the situation is different due to the fact that it is still under development. There are no simulated data, so we assumed that we observed an asterism without natural stars in the field of view. In this case, only laser guide stars were used, and random data was generated to train the network. This means there were 7200 input neurons for DRAGON. In Table 1 we can see a summary of the sizes of the different networks. 

## 4. Overview of Neural Network Frameworks

There is a long list of existing neural network frameworks [29], but the focus of this paper will be mainly on three of them: Caffe, Torch, and Theano. The reason to choose these frameworks is their popularity in the neural network community [30]. In addition, a neural network written in native C/CUDA was included in the comparison, with the aim of checking if training and execution times with the different frameworks can be further improved. 

### 4.1. Caffe

Caffe is a deep learning framework developed by the Berkeley Vision and Learning Center, and is released under the BSD 2-Clause license. It is mainly written in C/C++, and uses CUDA to speed up the execution on the GPU, with support for Nvidia Cuda Deep Neural Network Library (CuDNN) [44]. It has interfaces for Matlab, Python, and command line execution, which was used in this case. The used version had commit c2769c1.

### 4.2. Torch

Torch is a scientific computing framework with wide support for machine learning algorithms. It is written in Lua and C/CUDA to provide fast execution and includes the possibility of importing modules to complete and accelerate the system. It is mainly used and maintained by some large internet companies such as Google, Facebook, Twitter, etc. The used version had commit 0f873a8.

### 4.3. Theano

Theano is a Python library developed to help researchers in their deep learning investigations [45]. It is developed at the University of Montreal and uses some common Python libraries such as numpy and scipy. It also provides GPU support to speed up training and execution. The used version was v0.7.

### 4.4. C/CUDA

The purpose of this code is to be as light as possible, avoiding any unnecessary calculation or computation. The code is directly written in C and CUDA. For the execution process, we used the CUBLAS library to compute the matrix multiplications during the forward pass of the neural network. Also, the cuDNN library was employed to calculate the activation functions after each layer.

In the backpropagation process, we wrote CUDA kernels to calculate the error. We also employed CUBLAS to propagate the error backward through the different layers. During the computation, asynchronous data copies between central processing unit (CPU) and GPU memory were performed so that data transfer and computation could occur simultaneously. The version of CUDA used was CUDA 7.5 and cuDNN v3.

## 5. Experiment Description

To compare these frameworks, different networks were defined for the optical instruments described above. Two measurements were considered to evaluate their performance. Resulting training times under specific conditions and execution time of the network, which is crucial in adaptive optics systems, were compared.

The implementation of CARMEN in the different frameworks was done with the aim of getting the maximum efficiency of each framework and avoiding non-essential operations like shuffling the data during the training.

### 5.1. Training Benchmark

There are a large number of parameters to fit when training a neural network, although only a few of them directly affect the training time. To simplify the comparison, we will only vary those parameters that are relevant for the different systems.

One of the common characteristics to all networks is the backpropagation method. In every case, the Stochastic Gradient Descent (SGD) was used, with mini-batches and momentum, which was implemented in all the frameworks and the CUDA code. Two parameters are needed for this method: the learning rate and the momentum. Although these variables are crucial regarding the quality of the resulting network, they have no influence on the training times, so it was assumed that both are optimized to achieve the best possible result. We also have omitted from the paper any comparison using a validation set to check the quality of the training, because the same training method was used for all the frameworks, so it is a fair assumption that by making a good choice of hyperparameters, all them will provide very close results. 

Another critical parameter is the size of the training data. However, it is evident that in a training method based on mini-batches, time grows proportionally to the number of samples employed. Keeping this in mind, it is easy to calculate how much time a network will take to train when changing the training dataset. This idea makes the choice of the dataset size irrelevant for benchmarking purposes, although it is crucial for obtaining a good network. In this case, the size of the training data is specified in Table 1.

Three different networks were used, one per optical system, as shown in Table 1. The main parameters changed were the network size and the size of the mini-batch, in order to compare times. The number of samples in the mini-batch were 16, 32, 64, 128, and 256, as these sizes are used in other comparisons [30,32]. 

First, the initialized weights and bias were copied to the GPU’s video random access memory (VRAM) (VRAM: GPU Video RAM, RAM: CPU RAM), and the training dataset was loaded in the main CPU RAM before starting the training. Then, a timer was started and the program copied the first mini-batch from RAM to VRAM and then performed the calculation loop over the entire dataset. This operation was repeated for 20 epochs, timing each of them individually. This procedure enabled us to obtain an average time for each epoch, and more reliable results, making it possible to see if there were significant time variations among different epochs. The variability in the measurements was negligible, since their variances had values below 1%.

### 5.2. Execution Benchmark

The same networks defined in Table 1 were used in this case. However, there are some important differences with the training benchmark that should be detailed.

First, the net was fed with a single input, instead of using mini-batches, simulating what happens in a real telescope. As the execution program is intended to be integrated in the real-time control system, it is a fair assumption that all the variables are already initialized, and the weight matrices copied into the VRAM. 

Caffe does not allow us to integrate the code easily in a system if their parameters have been previously loaded and were omitted in the comparison. 

We cannot assume that the input data will be loaded into the main RAM, so we differentiated two cases: one with the data already loaded and a less ideal scenario where we have to read the information from the main disk. In the first one, we started the timer once the data was already loaded into the RAM and stopped it once the data was copied back from the GPU to the RAM. In the second case, the loading of input data from the solid state drive (SSD) was taken into account in the time measurement, as well as the time to copy data from the VRAM to the system RAM. Also, in this case, we timed the writing process from the RAM to the disk. 

Each input was found in a separate file, in h5 format [46], and the output was written to a separate file. In this case, it was not possible to measure Theano execution when the input was already loaded in RAM and not in VRAM since there is no explicit copy in the code between these two memories.

We fed the system with 10,000 inputs, one at a time, which allowed us to measure the execution time. We compared the average time of the different frameworks and determined whether there was any significant difference between them.

### 5.3. Experiment Equipment

The experiments were performed on a computer running on Ubuntu LTS 14.04.3, with an Intel Xeon CPU E5-1650 v3 @ 3.50 GHz, 128 Gb DDR4 memory, Nvidia GeForce GTX TitanX, and SSD hard drive. 

## 6. Results

In this section results obtained are shown, both in the training and execution process. The times are split to represent the different adaptive optics systems, and the possible explanations for the different results are analysed. 

### 6.1. CANARY-B1

**Figure 4:**
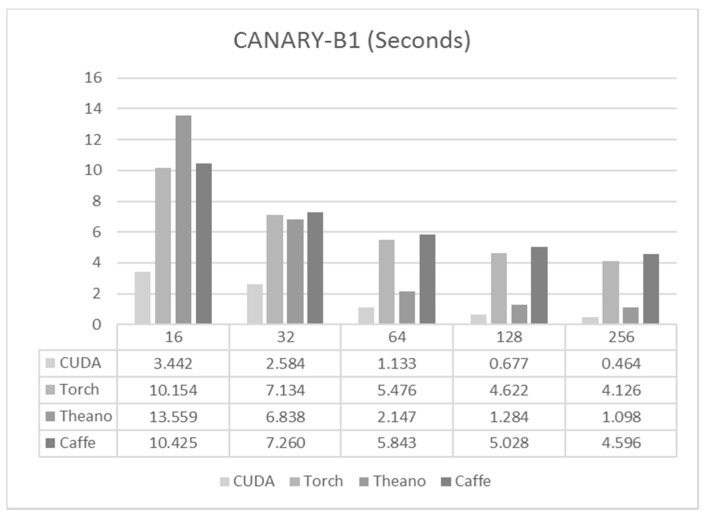
Training times per epoch for CANARY-B1.

As explained above, the network size was 216-216-72, and 350 k samples were used for training. As is shown in Figure 4, increasing the size of the batch had an obvious improvement on the training times, although it affected each framework in different ways.

For all batch sizes, the best results were obtained from the code written in C/CUDA, which was at least twice as fast as Theano. Caffe and Torch showed that although they were comparable to Theano for small batches, they were not able to reduce notably their training times when the batch size increased.

**Figure 5:**
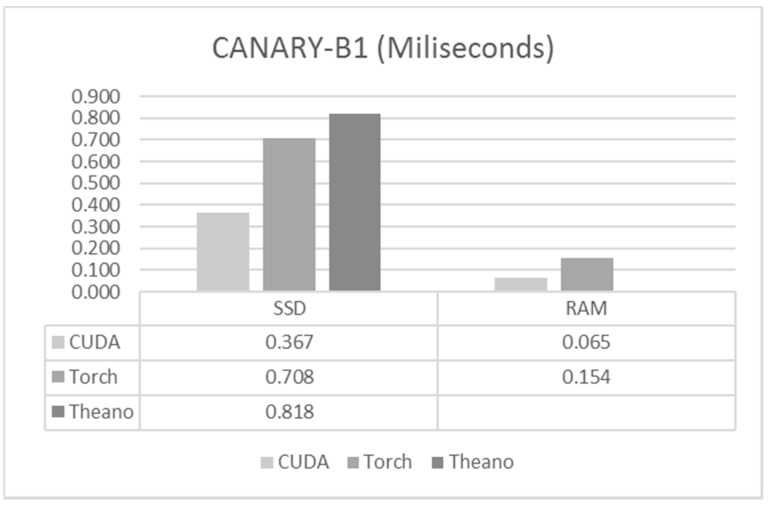
Execution times for CANARY-B1.

Execution times (forward propagation) had slightly different results and are shown in Figure 5. The code written in CUDA was still the fastest, being, again, twice as fast as the next one. Torch was the second fastest, with a small improvement over Theano. The difference between loading the data from the RAM versus loading it from the SSD was quite large, increasing the execution times by a factor of about five times.

### 6.2. CANARY-C2

In the case of CANARY-C2, the network size was 1152-1152-288 and used 1.5 M training samples. In this case, the reduction of batch size had more impact in reducing the training time due to the larger size of the network.

**Figure 6:**
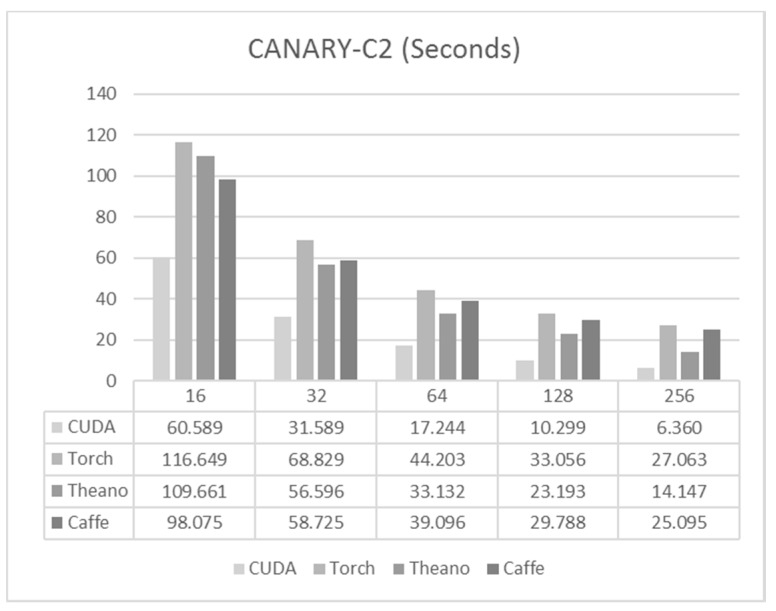
Training times per epoch for CANARY-C2.

Again, the code written in C/CUDA was the fastest for all the cases as shown in Figure 6, being about two times faster than the other frameworks. For this net, Caffe showed good performance, but was not able to keep reducing its training time when the batch size was increased. In the case of Theano, a good performance was obtained for large batch sizes, but performance got worse as the batch size decreased. In this scenario, Torch had the worst training times, although they were not far from the times obtained by Caffe.

**Figure 7:**
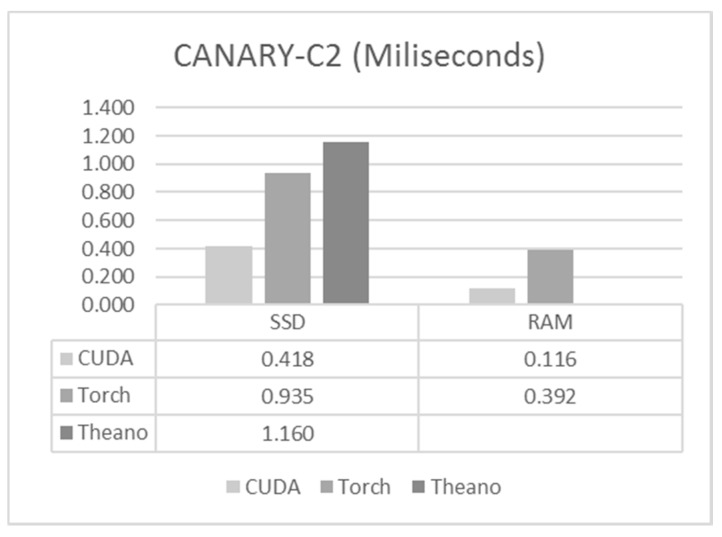
Execution times for CANARY-C2.

For execution times we have a very similar situation to that which was observed for the CANARY-B1 case, as we can see in Figure 7. C/CUDA achieved the best execution time in both cases, followed by Torch, with Theano in the last position. There was also a significant difference between loading the data from the SSD and when we had the data already loaded into RAM, as was expected.

### 6.3. DRAGON

**Figure 8:**
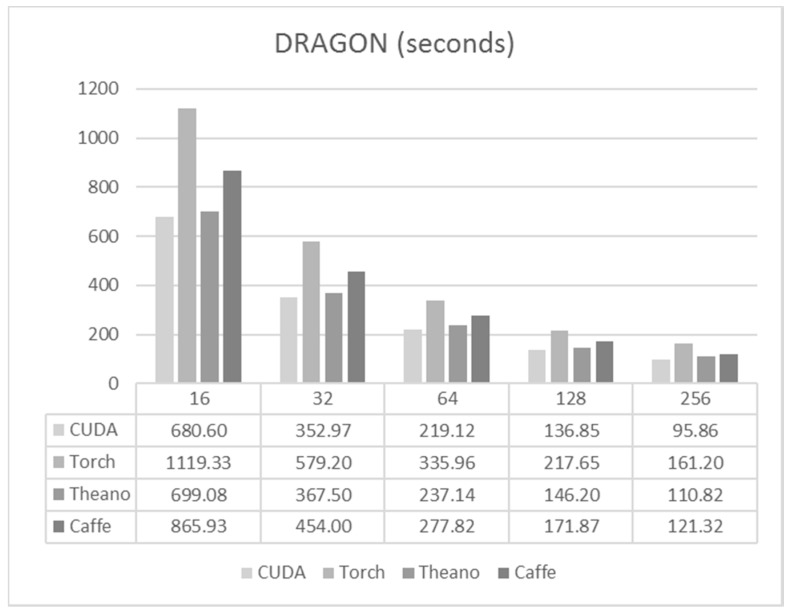
Training times per epoch for DRAGON.

The largest network was employed for this case, with 7200-7200-1800 neurons and 1 M samples for training. For this case, training times were much more similar for the different frameworks, and the reduction due to the batch size was even larger, as is shown in Figure 8.

Again, C/CUDA code managed to obtain the best results. However, in this case, Theano got very close to the times shown by C/CUDA. Also, the differences between Caffe and Torch were much smaller than in the case of the previous nets. 

**Figure 9:**
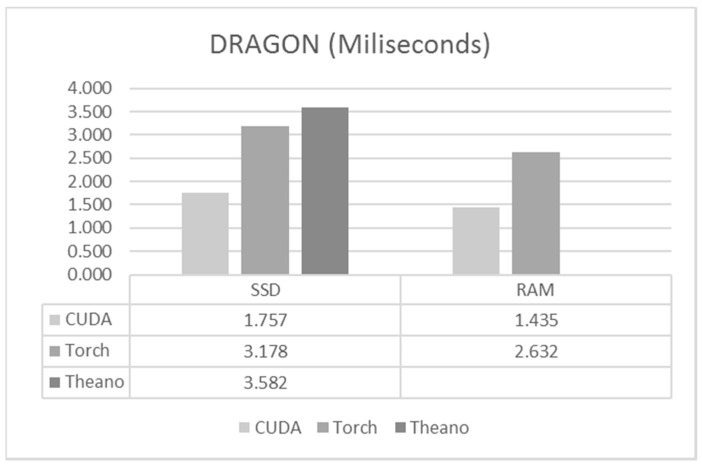
Execution times for DRAGON.

For forward propagation, similar results to the previous cases were obtained for the DRAGON system. In this case (Figure 9) the difference between the frameworks was even smaller. Also, loading the data from the disk was not much slower than loading data from the RAM.

### 6.4. Discussion

As we can see in the previous figures, a code written directly in C/CUDA is the fastest option for all these cases. This is expected behaviour, since most of the frameworks are designed to deal with much more complex neural networks.

We can see that, especially for small networks, the difference between different methods could be huge. Although training a small net like CANARY-B1 could take only a few minutes with any of the analysed frameworks, it is interesting to reduce the times as much as possible, because on-line training could be an interesting option. In real telescopes, atmospheric conditions change over time periods as small as a few seconds, and having a framework which can re-train the network with a different set of data could help to maintain AO performance.

For larger networks, the difference in performance is smaller, especially with DRAGON, where the difference between Theano and C/CUDA is almost zero. This behaviour can be explained by how the computation time is spent. In small networks, any overhead introduced by the framework could make a big difference between the different systems. However, as the size increases, more time is spent on computing the outputs and backpropagating the error, so the impact of the possible overhead is much smaller. Another explanation for the time difference between the frameworks is that they used different libraries (CUBLAS, Thrust) to compute the values.

For neural network execution, the C/CUDA code is the fastest solution for all the optical systems. In adaptive optics for large telescopes, we usually receive a new input approximately every 1–4 ms. This time is usually taken as the limit to provide an output to the system due to the rapid changes in the atmospheric turbulence. In the case of DRAGON, only C/CUDA is able to provide an output below a 2 ms limit (corresponding to an AO system update rate of 500 Hz). However, even for small networks, the goal should be to have an output as fast as possible, so we can achieve the smallest possible delay between observation and correction, leading to the best performance

## 7. Conclusions and Future Lines

We have analysed different frameworks for training and executing of a tomographic wave-front reconstructor based on artificial neural networks, as used by CARMEN. In addition, we have compared the execution and training times of the networks deployed using these frameworks to neural networks programmed by the authors using C/CUDA code and more low-level libraries such as CUBLAS. Writing the code directly in C/CUDA showed the best results in every case, which makes it the proper solution for the studied systems. 

Nevertheless, we consider the analysis of how the different neural network frameworks can perform as a good start to the study of how more complex neural networks can improve the performance of CARMEN as a tomographic reconstructor. Using convolutional neural networks [47] or recurrent networks [48] can provide some boost regarding the quality of the reconstruction. Although this could be done programming the code directly in C/CUDA, the use of any of the existing frameworks can definitely make it easier to find a new neural network architecture that could help CARMEN.

Finally, we still have to address some possible challenges in the near future. It is expected that the number of inputs and outputs of AO systems will significantly grow over the next years as ELT systems come online [24]. This will increase the time required for training the network. Moreover, we will have to face issues regarding the amount of GPU memory needed to deal with larger weight matrices. A possible workaround for this is to train and execute the network, using multi-GPU systems, where the computation and the memory can be split across different graphics cards. Some of the studied frameworks offer the possibility to train the network by splitting the batches between different GPUs, which can help to reduce the training times. However, it would be interesting to study if it is possible to divide the weight matrices across multiple graphics cards, avoiding problems with the amount of VRAM available.

## Figures and Tables

**Table 1:** Adaptive optics and neural networks summary.

Name	Network Size	Training Data (Number of Samples)
CANARY-B1	216-216-72	350,000
CANARY-C2	1152-1152-288	1,500,000
DRAGON	7200-7200-1800	1,000,000
